# Supplementary figures and images for: Contribution of Stenotrophomonas maltophilia MfsC transporter to protection against diamide and the regulation of its expression by the diamide responsive repressor DitR
Source: PLoS One. 2022 Aug 1;17(8):e0272388. doi: 10.1371/journal.pone.0272388 (PMC9342713; doi:10.1371/journal.pone.0272388)

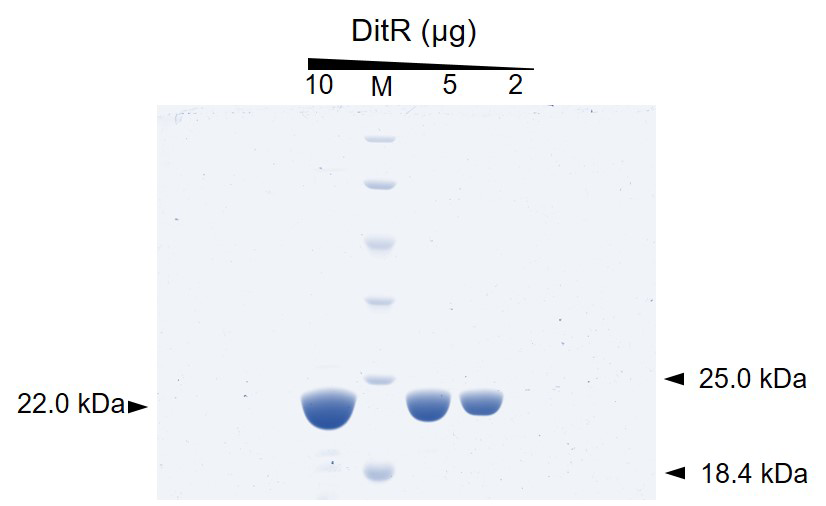

Supplement: S1 Fig — The coomassie blue staining of DitR proteins (2, 5 and 10 μg) after purification and separation by 12.5% SDS-PAGE. M represents protein molecular weight markers. (TIF) [file pone.0272388.s001.tif]

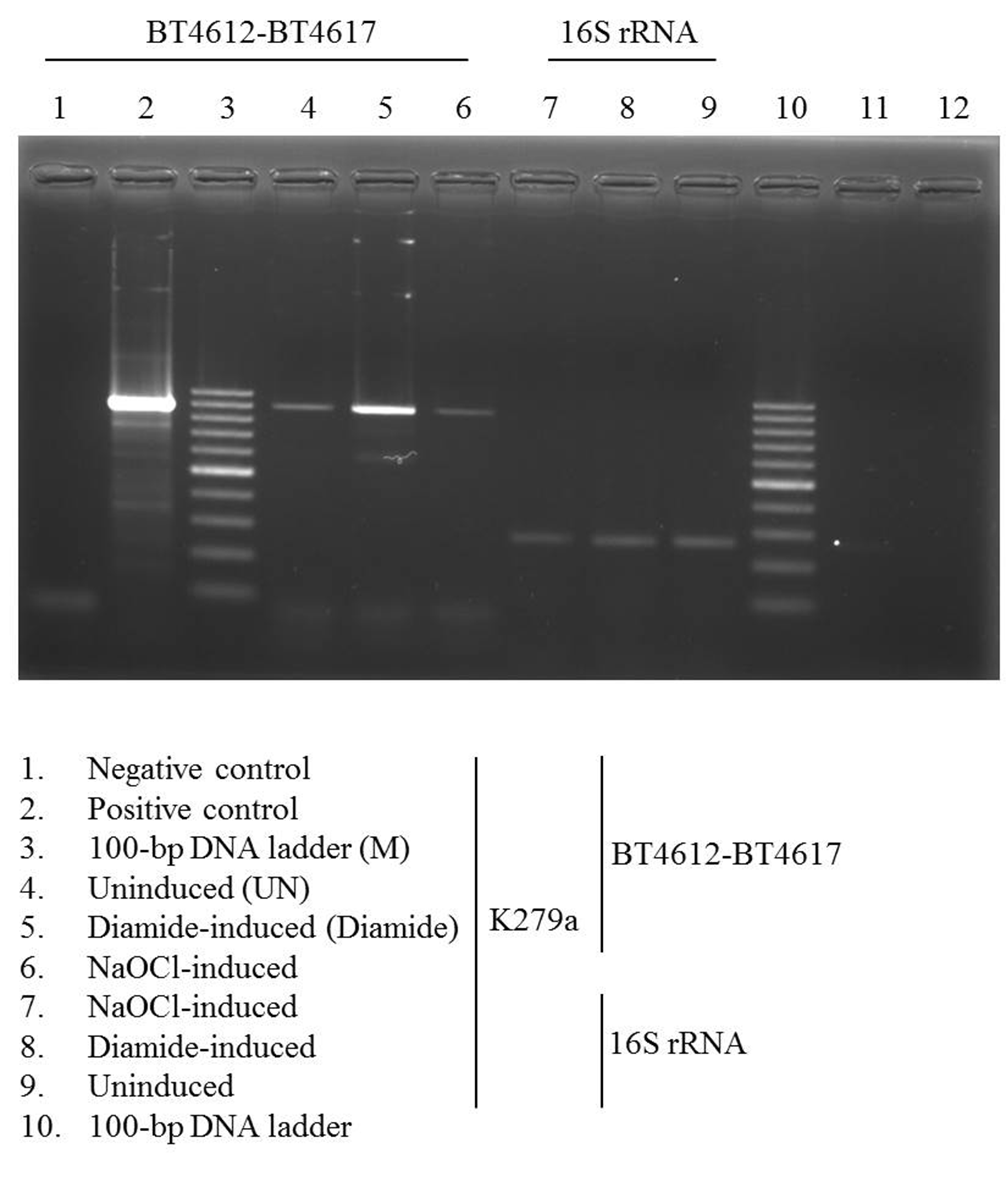

Supplement: S2 Fig — (TIF) [file pone.0272388.s002.tif]

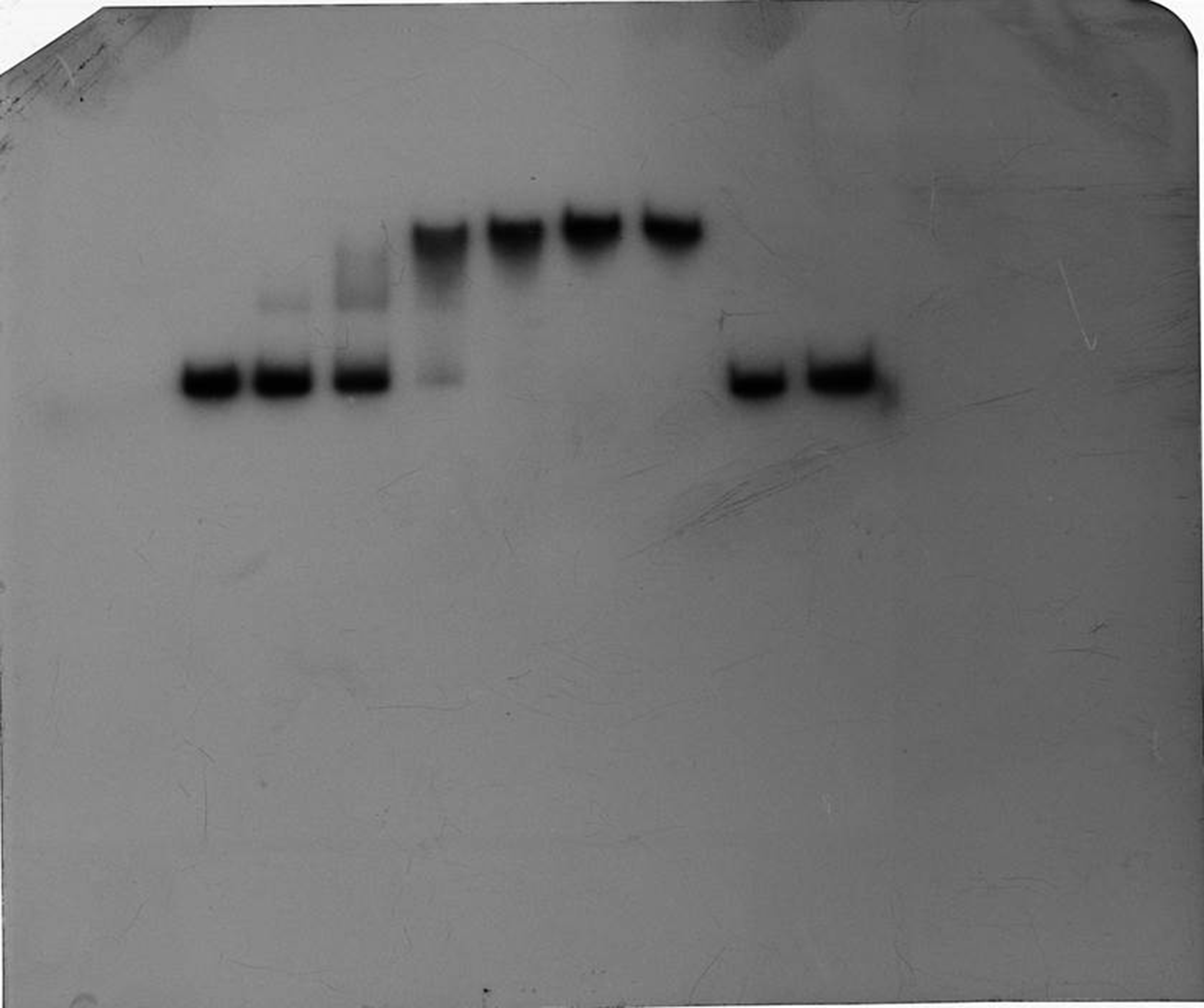

Supplement: S3 Fig — (TIF) [file pone.0272388.s003.tif]

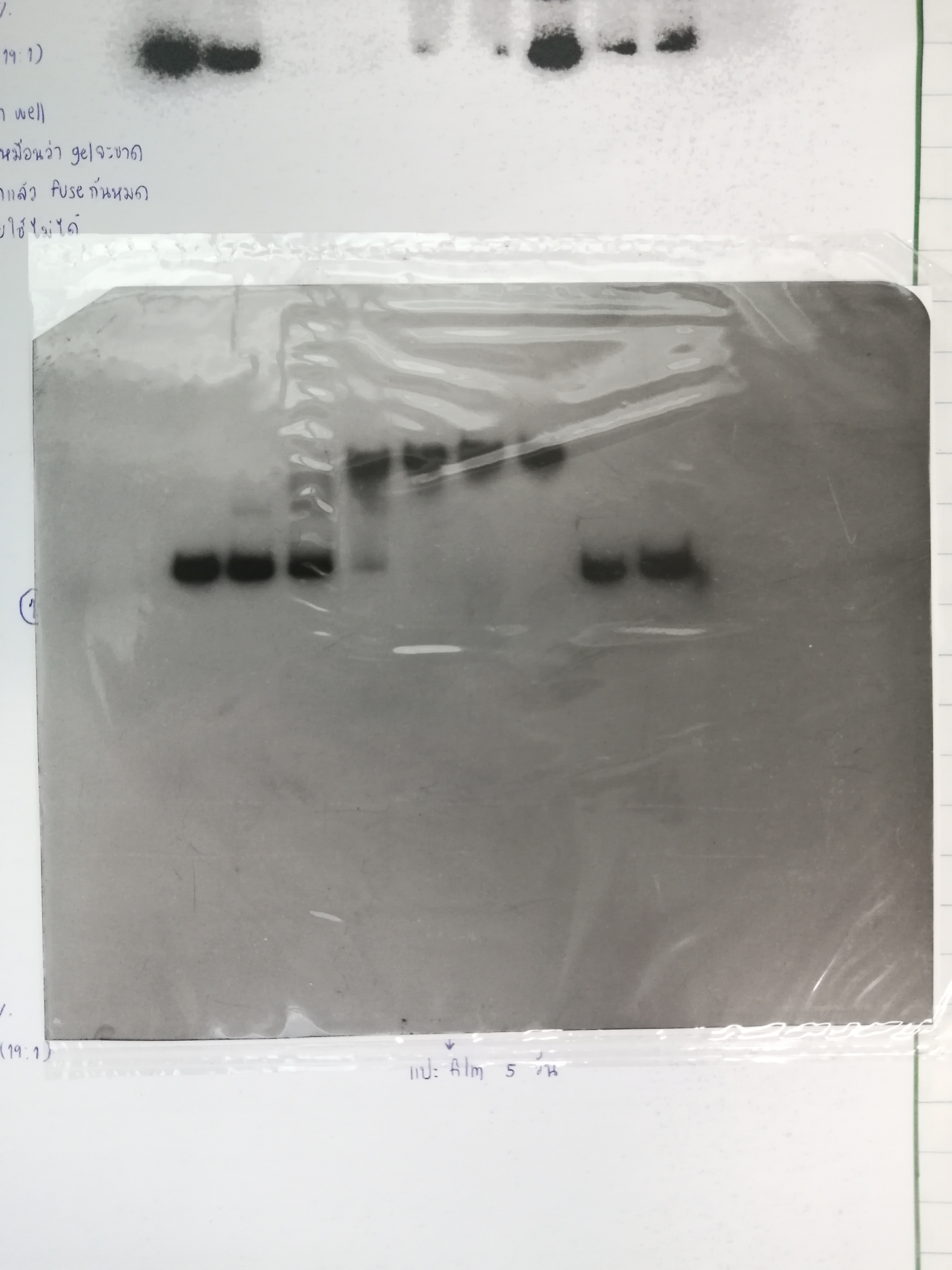

Supplement: S4 Fig — (TIF) [file pone.0272388.s004.tif]

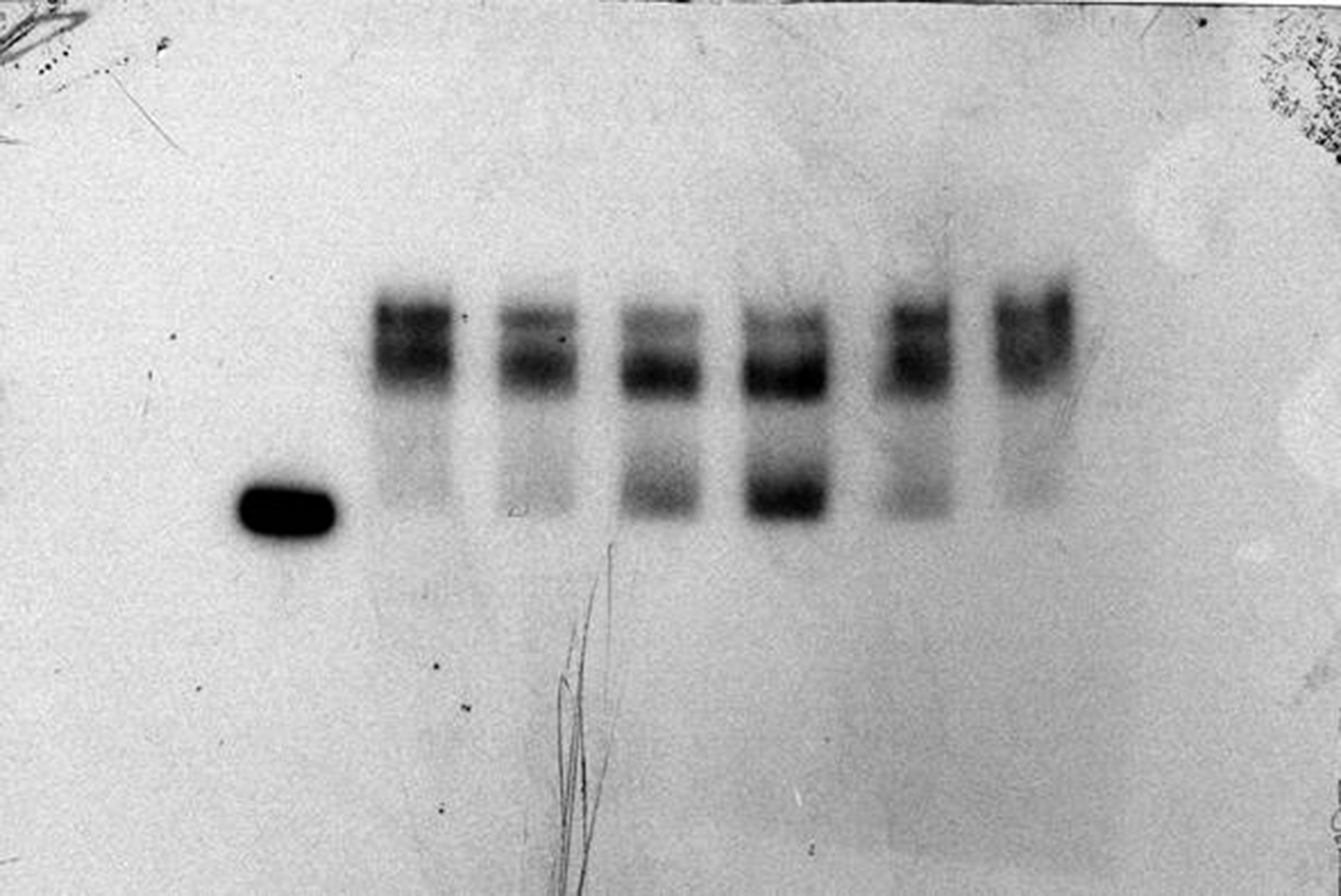

Supplement: S5 Fig — (TIF) [file pone.0272388.s005.tif]

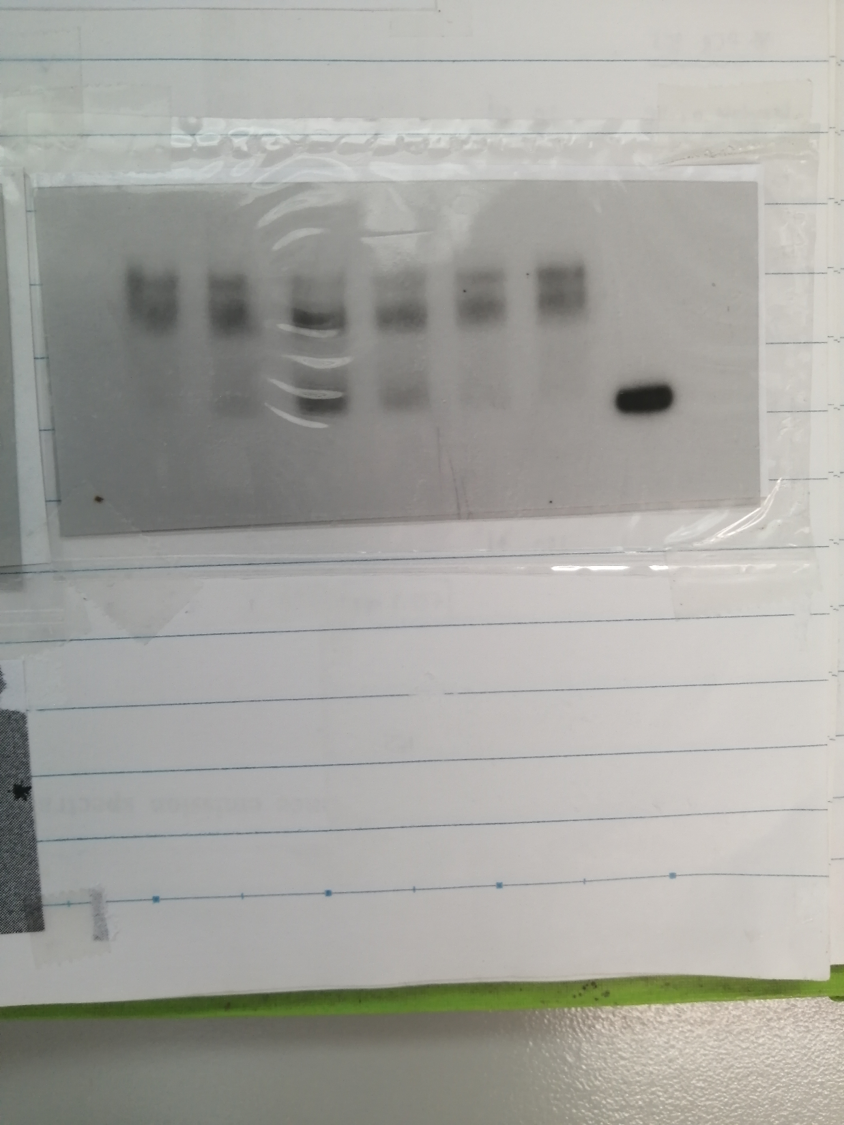

Supplement: S6 Fig — (TIF) [file pone.0272388.s006.tif]

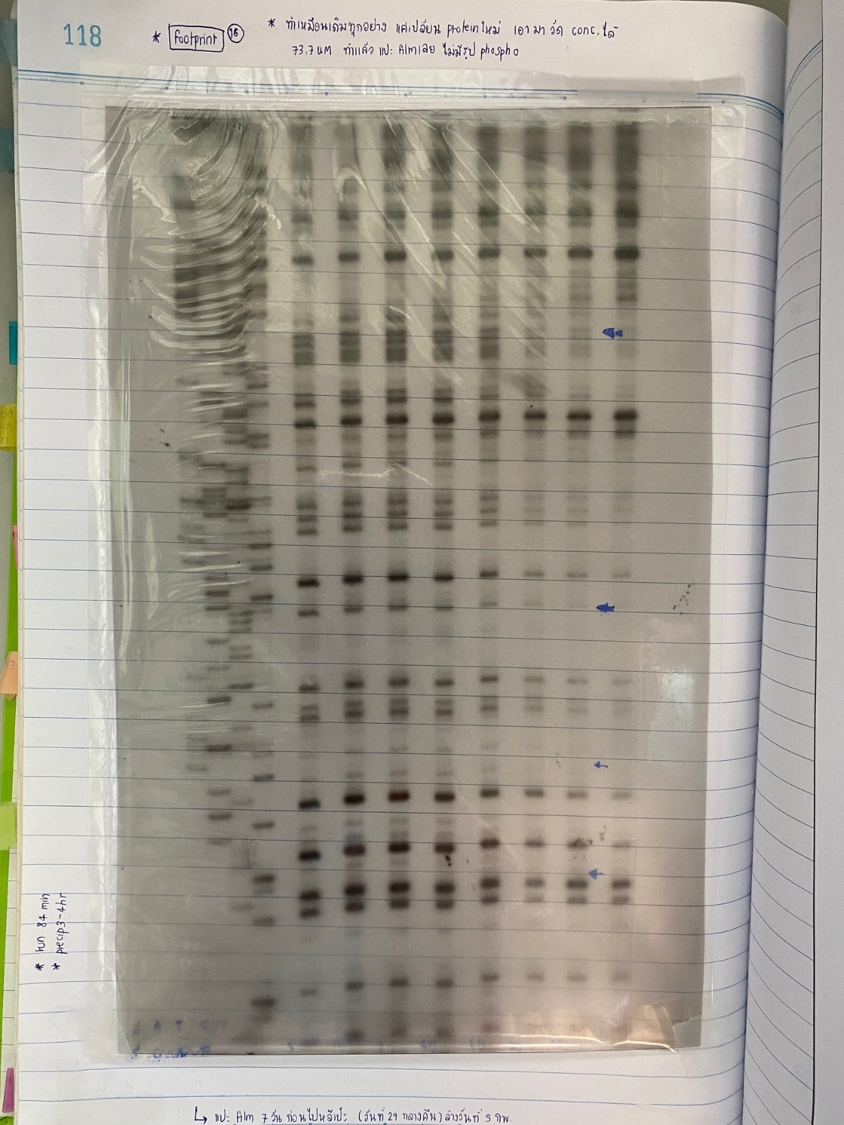

Supplement: S7 Fig — (TIF) [file pone.0272388.s007.tif]

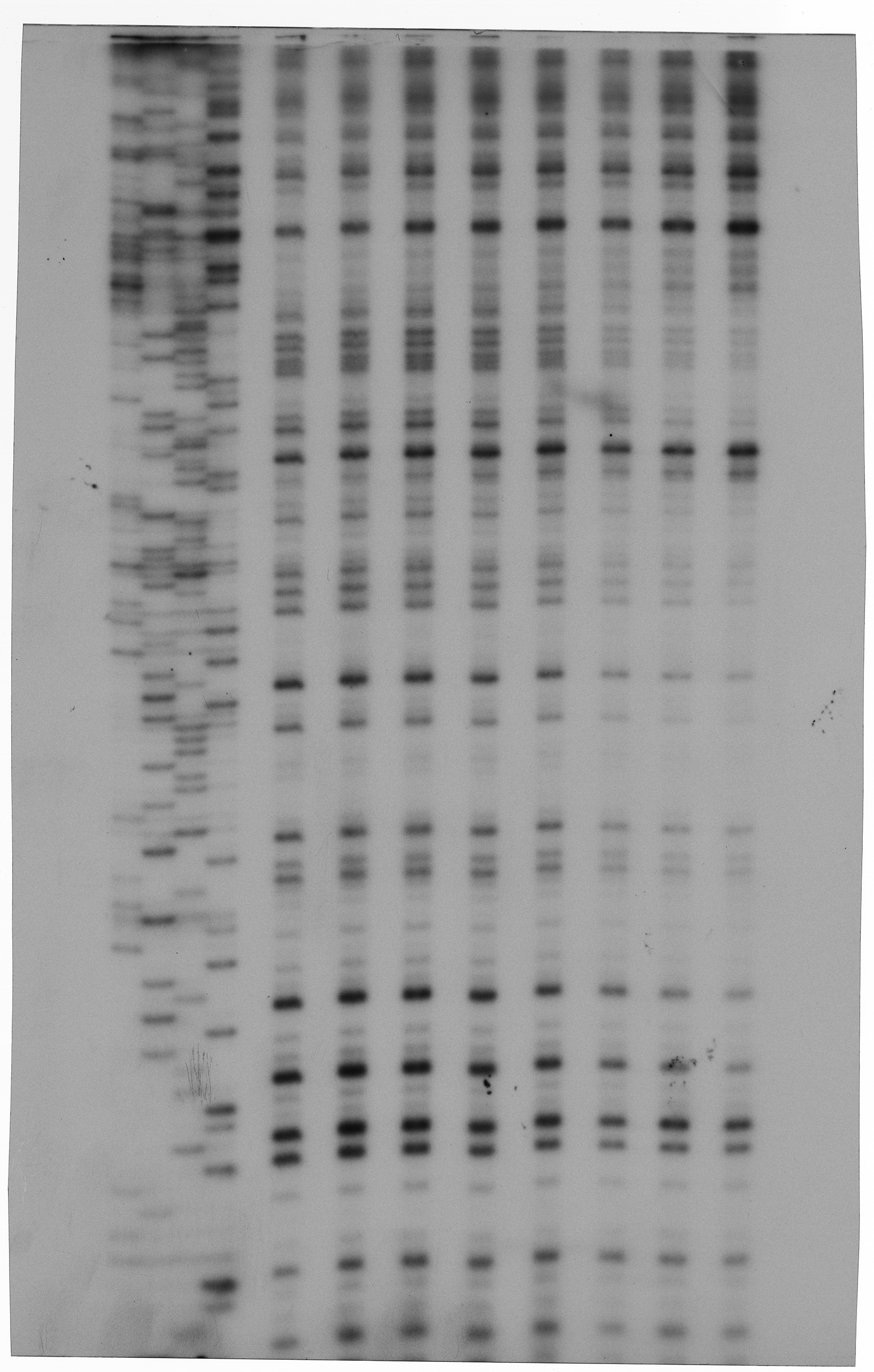

Supplement: S8 Fig — (TIF) [file pone.0272388.s008.tif]

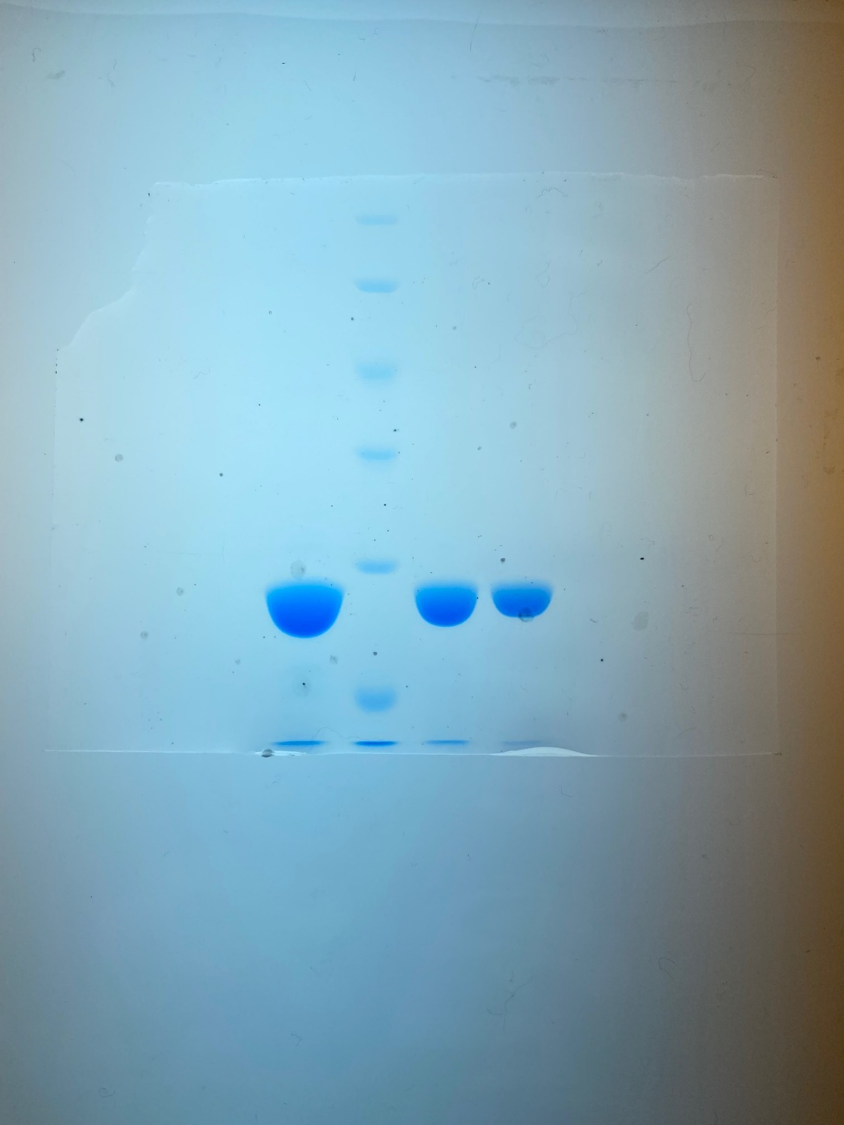

Supplement: S9 Fig — (TIF) [file pone.0272388.s009.tif]
