# Supplementary material for: Contribution of Stenotrophomonas maltophilia MfsC transporter to protection against diamide and the regulation of its expression by the diamide responsive repressor DitR
Source: PLoS One. 2022 Aug 1;17(8):e0272388. doi: 10.1371/journal.pone.0272388 (PMC9342713; doi:10.1371/journal.pone.0272388)

Raw-image of Figure 4B
Experiments: end point RT-PCR from RNA samples
Method used to capture image: Photograph ubder UV-light

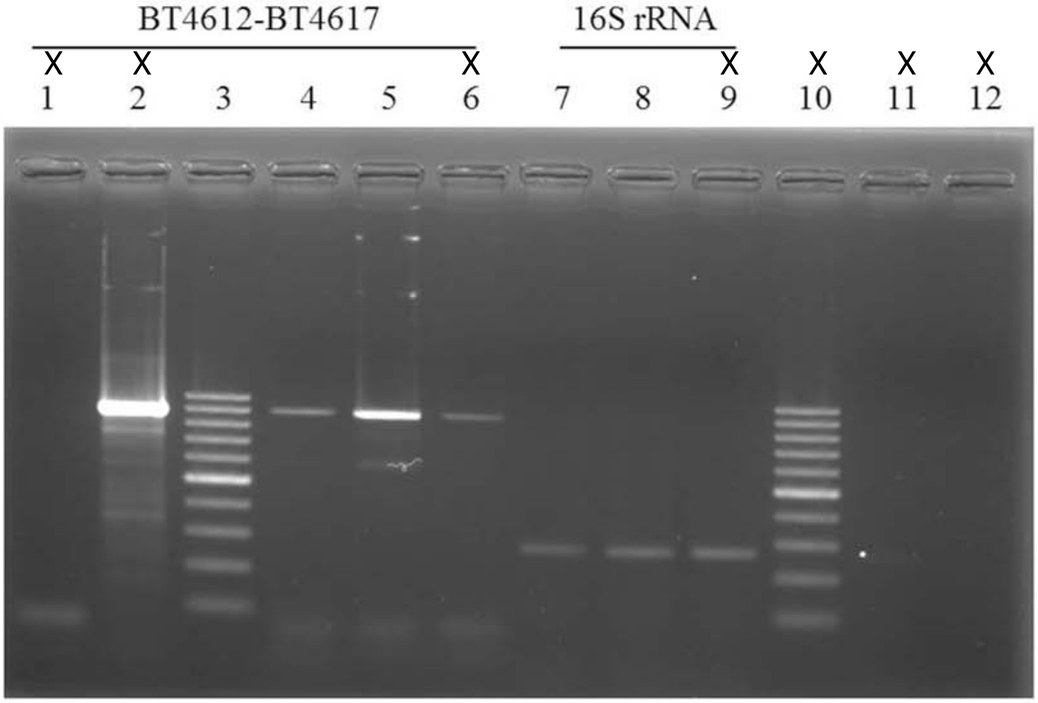

1.	Negative control	K279a	BT4612-BT4617
2.	Positive control		
3.	100-bp DNA ladder (M)		
4.	Uninduced (UN)		
5.	Diamide-induced (Diamide)		
6.	NaOCl-induced		
7.	NaOCl-induced		16S rRNA
8.	Diamide-induced		
9.	Uninduced		
10.	100-bp DNA ladder		

Raw-image of Figure 7A

Experiment: Gel shift assay
Method used to capture image: scanning of the X-ray film

(all lanes are uesd in the manuscript’s main Figure 7A.)

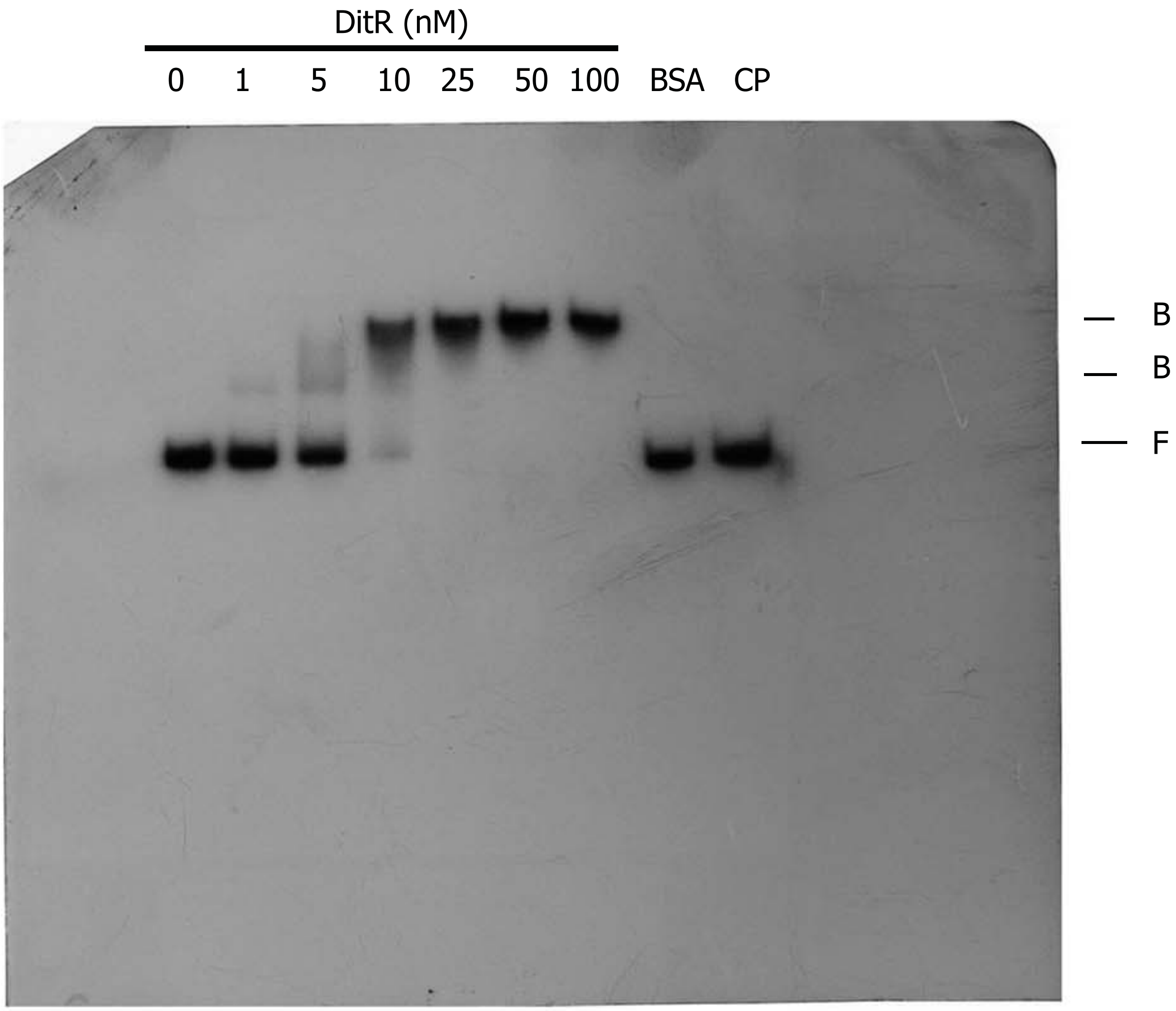

Raw-image of Figure 7B
Experiments: Gel shift assay
Method used to capture image: scanning of the X-ray film
(all lanes are used in the manuscript’s main Figure 7B.)

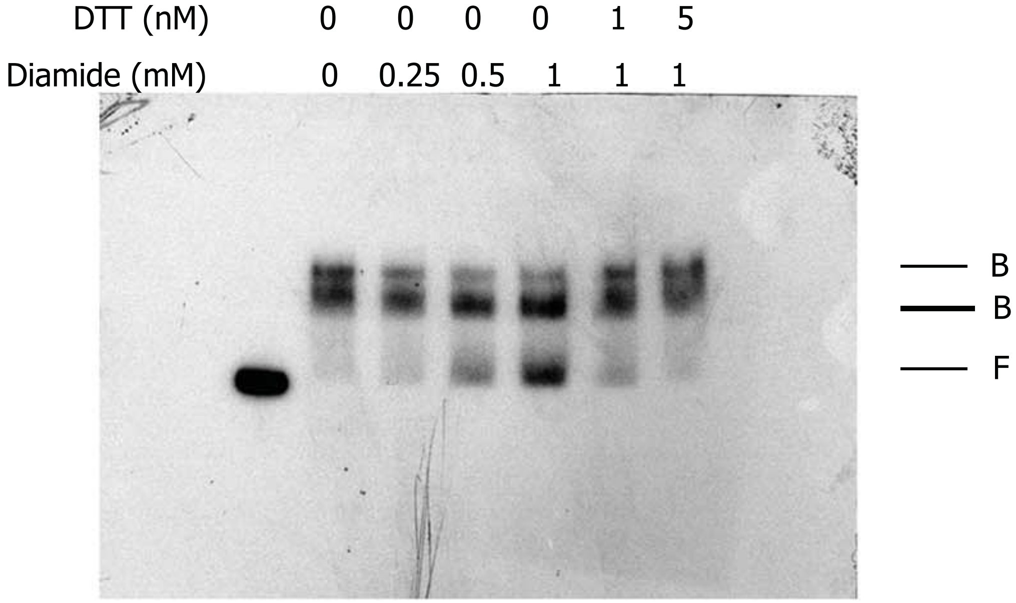

Raw-image of Figure 9B
Experiments: DNaseI footprinting assay
Method used to capture image: scanning from the X-ray film
(all lanes are used in the manuscript's main Figure 9B.)

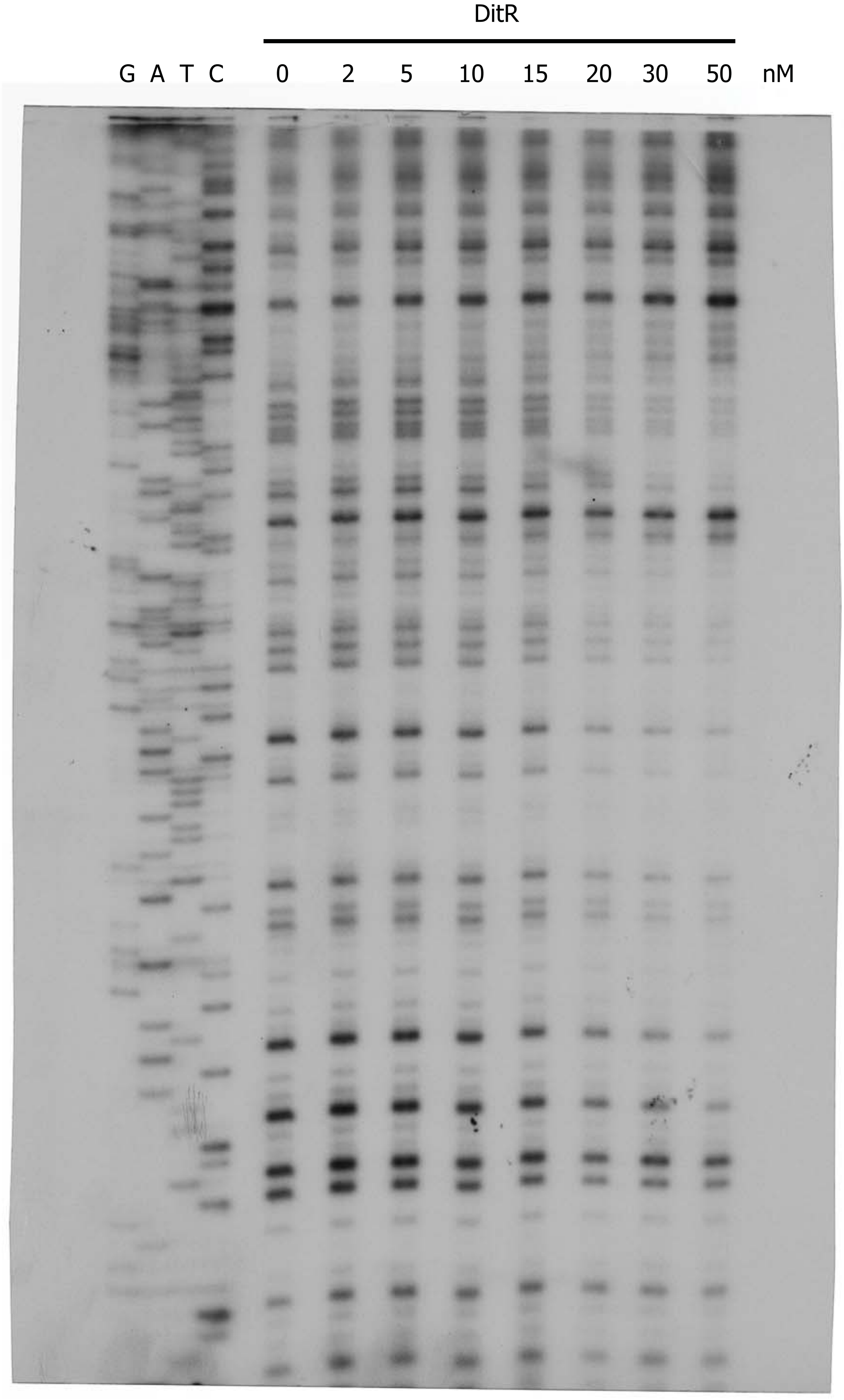

Raw-image of S1 Fig
Experiments: SDS-PAGE of purified DitR protein
Method used to capture image: photograph under visible light

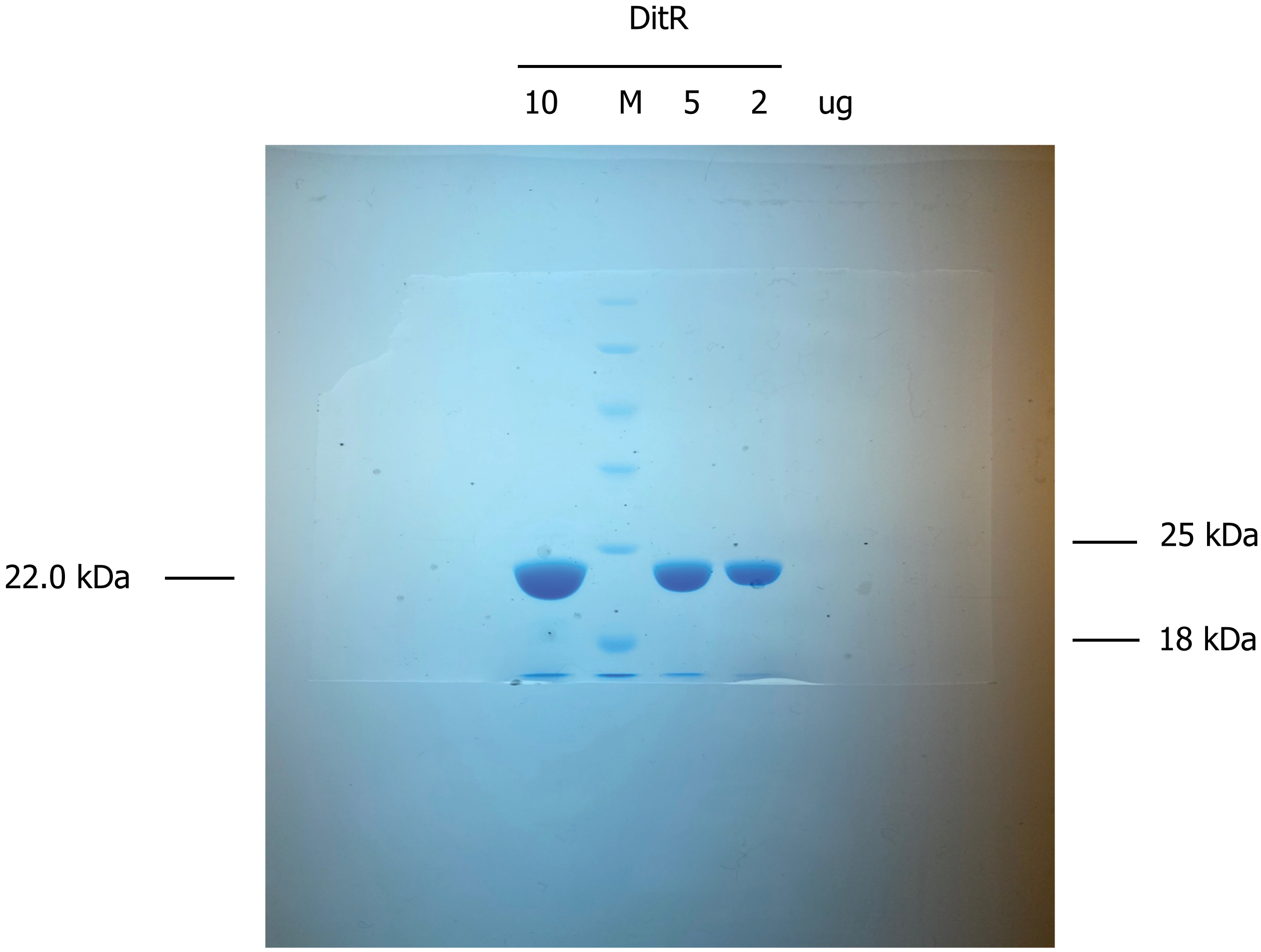

Supplement: S1 Raw images — (PDF) [file pone.0272388.s014.pdf]
