# Supplementary material for: Contribution of Stenotrophomonas maltophilia MfsC transporter to protection against diamide and the regulation of its expression by the diamide responsive repressor DitR
Source: PLoS One. 2022 Aug 1;17(8):e0272388. doi: 10.1371/journal.pone.0272388 (PMC9342713; doi:10.1371/journal.pone.0272388)
Supplement: Attachment — Submitted filename: Responses to reviewers.docx [file pone.0272388.s015.docx]

**Point-by-point Response to Reviewers**

PONE-D-22-09616

Contribution of Stenotrophomonas maltophilia MfsC transporter to protection against diamide and the regulation of its expression by the diamide responsive repressor DitR

PLOS ONE

Reviewer #1: The manuscript presented characterization of MfsC transporter to protection against diamide and the regulation of its expression by the diamide responsive repressor DitR in Stenotrophomonas maltophilia. Before the paper could be accepted for publication in PLOS ONE, the authors need make revisions as follow:

1. What’s the function of MfsB? What’s the relationship between MfsB and MfsC? Please add discussion.

Reply: According to our current data, the function of MfsB is still unknown. The Δ*mfsB* mutant showed no altered phenotypes against all tested oxidants, antibiotics and biocides. *mfsB* was co-transcribed with *mfsC* and its expression could be induced by diamide. We have added discussion about this *mfsB* as “Although *mfsB* was co-transcribed with *mfsC* in an diamide-inducible manner, it seems likely that MfsB has no role in the protection of *S. maltophilia* against all tested antibiotics and oxidants (Fig 2). The precise physiological role of *S. maltophilia* MfsB is as yet unknown and required further investigation.” (line 344-347)

Reviewer #2: This is a very nice characterization of a transporter operon and its regulator that mediated resistance to diamide in Stenotrophomonas maltophilia (Steno). The authors show deletion of mfsC leads to increased sensitivity to diamide and that the regulator smlt0547 (named ditR here) is a repressor (based on deletion, complementation, and EMSA behavior) that binds the mfsC operon promoter (shown by EMSA and DNase footprinting) and is released by the presence of diamide (shown by EMSA) resulting in operon expression specific to diamide presence (qRT-PCR and reporter assays). Release by diamide is reversible by DTT treatment and loss of conserved reactive cysteines in DitR result in a diamide-insensitive and thus constitutively repressive protein (shown by reporter expression). These findings are related to the field via appropriate discussion and phylogenetic analyses. I think this is a really nice story and really enjoyed reading it. I have a few minor things to suggest or to be clarified, followed by some suggested text edits to make the manuscript more clear.

1. For the various compounds, particularly the oxidants, why were these concentrations chosen? It seems as if diamide is used at the highest concentration and one could argue that the repressor could be responsive and/or transporter active if the other compounds were present at higher concentrations. I do not think this is the case, but an explanation of why these concentrations were chosen would be very helpful.

Reply: The oxidant concentrations that we used in expression analysis are the sub-minimal inhibitory concentrations (sub-MIC). We have added this statement on line 187.

2. Statistics: For all of the comparisons in which there are more than two groups (all of them) any t-tests need to be corrected for multiple comparisons by an appropriate post-test. For Fig 3 it would be a Dunnett’s post-test with WT as the comparator tested within each compound. For Fig 4 it would be a Dunnett’s post-test with untreated as the comparator. I don’t think this increased statistical rigor will change the conclusions but use of multiple-comparison corrected stats is important.

Reply: Thank you for this valuable suggestion. We have changed statistical analysis to Dunnett’s post-test as suggestions and we found that it did not affect conclusions in both Fig 3 and Fig 4.

3. Figure suggestions: Fig 1 is unnecessary, the description in the text is sufficient. For the phylogenetic trees, I would prefer plotting the trees with quantitative branch lengths (actual branch lengths with an amino-acid difference scale) so that relative similarity is visually represented.

Reply: Fig 1 has been removed as suggestion. The branch length of the phylogenetic trees in Fig. 2 (now Fig 1) and Fig 7 (now Fig 6) has been changed to an actual branch length to visually present the differences as suggestion.

4. Methods: Line 97: I find it really hard to believe that a bunch of molecular biology was conducted according to a 21 year old method manual without the use of any kits or any deviations from those published methods. Please expand detail as needed. Line 110: Which plasmid was the cre recombinase expressed from and how was the strain cured of that plasmid?

Reply: Thank you for your comments. We did use some kits for plasmid preparation and extraction of DNA fragments from agarose gel. We have mentioned this on line 100-102.

The Cre-recombinase protein was expressed from pCM157 plasmid. We have added more detail about the excision of Gm^r^ cassette and curing of pCM157 as “To excise the Gm^r^ marker, pCM157 [15] expressing Cre-recombinase was introduced into the putative mutant and the resultant unmarked Δ*mfsB* mutants obtained from Cre-mediated *loxP* recombination were screened for the gentamicin sensitive phenotype. The plasmid pCM157 (carrying tetracycline-resistant gene) was cured by subculturing the strains on plates without antibiotic supplementation and screened for the tetracycline-sensitive colonies.” (line 111-116).

5. This is really just a comment to improve future work. Having myself tried lacZ in Steno many times, it really is not a great reporter and seems to be due to poor protein folding. The authors have appropriately used a Unit based measure, as these numbers would look ridiculously low if the common Miller unit were used (1 Bgal unit in this manuscript would be 0.002 Miller Units, if my calculations are correct). I don’t think anything needs to change for this manuscript, but I would suggest using a better reporter for Steno in the future. XylE works very well as an enzymatic reporter and mScarlet works really well as a fluorescent reporter.

Reply: We appreciate your valuable suggestions and it may be useful for our future works.

6. Text edit suggestions:

Line 25: change ‘transports’ to ‘transporters’

Reply: We have edited as suggestion.

Line 44: edit first sentence to- “*Stenotrophomonas maltophilia* is a Gram-negative, rod-shaped, aerobic bacterium, which is ubiquitous in the aqueous environment and soil.”

Reply: We have edited as suggestion.

Line 50: edit start of sentence to- “Efflux transporters are recognized…”

Reply: We have edited as suggestion.

Line 52: Since the assumption of kingdom number keeps changing, I suggest using “all domains of life” instead of numbering the kingdoms.

Reply: We have edited as suggestion.

Line 57: change ‘several’ to ‘many’ and replace ‘however’ with ‘and’

Reply: We have edited as suggestion.

Line 82: suggest “Here, we are interested in the MFS transporters because of their…”

Reply: We have edited as suggestion.

Line 83: change ‘gene’ to ‘transcriptional’

Reply: We have edited as suggestion.

Line 86: Suggest putting the smlt gene number for DitR and stating ‘… which we have named DitR’

Reply: We have edited as suggestion.

Line 351: change ‘direction’ to ‘orientation’

Reply: We have edited as suggestion.
